# Cloning of quantitative trait genes from rice reveals conservation and divergence of photoperiod flowering pathways in Arabidopsis and rice

**DOI:** 10.3389/fpls.2014.00193

**Published:** 2014-05-13

**Authors:** Kazuki Matsubara, Kiyosumi Hori, Eri Ogiso-Tanaka, Masahiro Yano

**Affiliations:** ^1^NARO Institute of Crop ScienceTsukuba, Japan; ^2^Agrogenomics Research Center, National Institute of Agrobiological SciencesTsukuba, Japan

**Keywords:** breeding, natural variation, photoperiodic flowering, quantitative trait gene, rice

## Abstract

Flowering time in rice (*Oryza sativa* L.) is determined primarily by daylength (photoperiod), and natural variation in flowering time is due to quantitative trait loci involved in photoperiodic flowering. To date, genetic analysis of natural variants in rice flowering time has resulted in the positional cloning of at least 12 quantitative trait genes (QTGs), including our recently cloned QTGs, *Hd17*, and *Hd16*. The QTGs have been assigned to specific photoperiodic flowering pathways. Among them, 9 have homologs in the *Arabidopsis* genome, whereas it was evident that there are differences in the pathways between rice and *Arabidopsis*, such that the rice *Ghd7*–*Ehd1*–*Hd3a*/*RFT1* pathway modulated by *Hd16* is not present in *Arabidopsis*. In this review, we describe QTGs underlying natural variation in rice flowering time. Additionally, we discuss the implications of the variation in adaptive divergence and its importance in rice breeding.

## Introduction

Photoperiodic flowering is one of the most important responses of plants to their environment (Thomas and Vince-Prue, 1997). In the last two decades, molecular genetics demonstrated that external light signals perceived by photoreceptors induce florigens (flowering signals). This process is regulated in part by the circadian clock, and promotes flowering in response to favorable daylength in both monocots and eudicots (reviewed by Andrés and Coupland, 2012).

Rice is a short-day (SD) plant, i.e., flowering is accelerated under SD conditions. Natural variation in rice flowering time is generated mainly by quantitative trait genes (QTGs) involved in photoperiod pathways, unlike in other cereals (such as wheat and barley) and *Arabidopsis thaliana*, which also need to be subjected to low temperature (vernalization requirement) (reviewed by Greenup et al., 2009; Tsuji et al., 2011; Bentley et al., 2013; Brambilla and Fornara, 2013; Itoh and Izawa, 2013).

**Figure 1:**
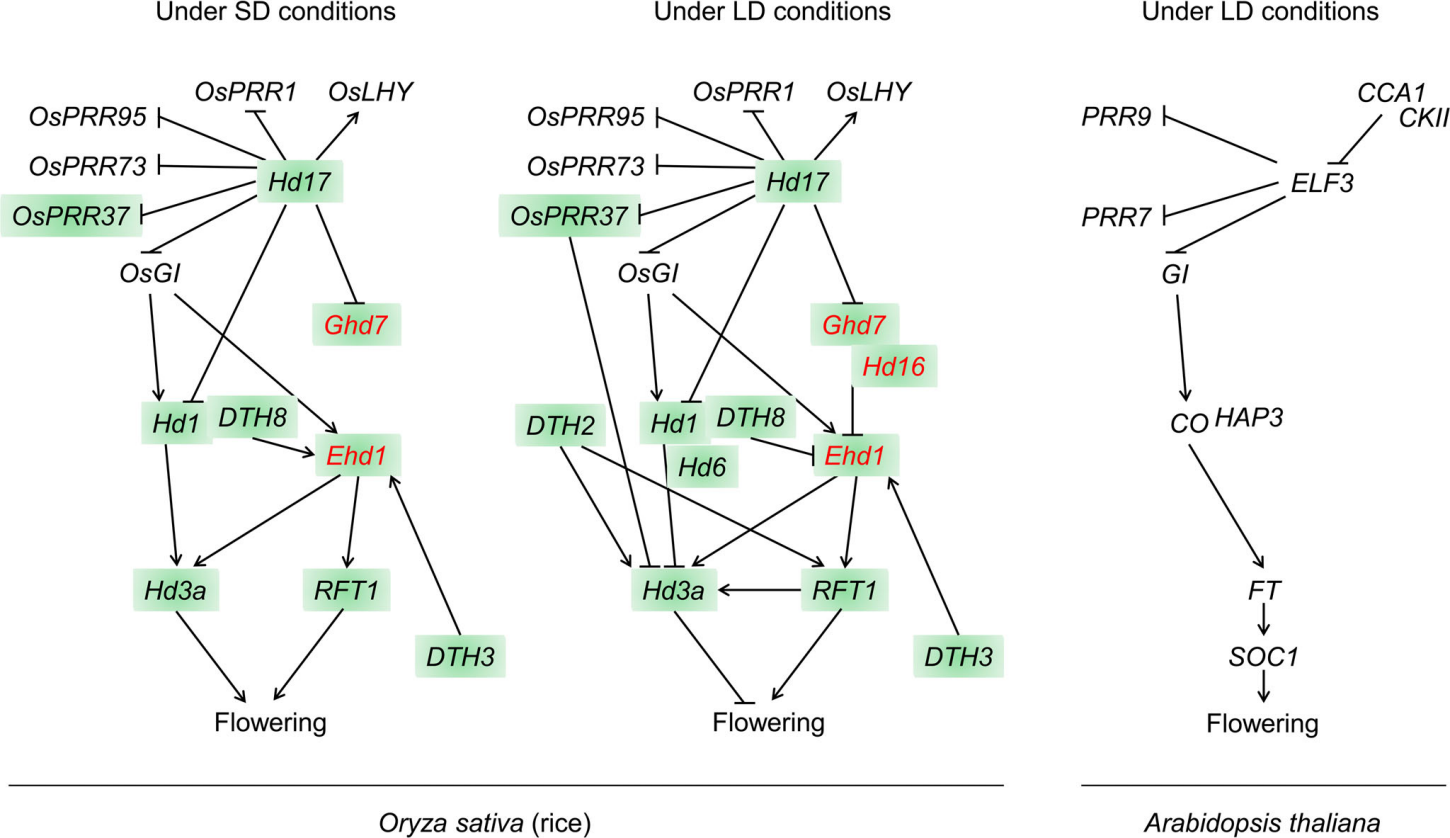
**Schematic representation of roles of QTGs within the photoperiodic flowering pathways in rice and *Arabidopsis thaliana***. QTGs that are marked in red are not present in *Arabidopsis*. QTGs that were cloned by QTL analysis of natural variation are highlighted. Arrow heads, up-regulation; Bars, down-regulation.

To date, at least 12 QTGs, which belong to the two independent flowering pathways, have been mapped and cloned through quantitative trait locus (QTL) analysis of natural variation in flowering time (Figure 1 and Table 1). In this review, we describe the molecular basis of QTGs underlying natural variation in rice flowering time and discuss the implications on adaptive divergence and consequences for breeding.

**Table 1:** **QTGs underlying natural variation in flowering time of rice**.

**Gene symbol**	**Full name**	**Locus ID**	**Synonym**	**Effect on flowering**	***Arabidopsis* homolog**	**Note**	**References**
*Hd1*	*Heading date 1*	Os06g0275000		SD promotion/LD repression	*CO*	B-box zinc-finger protein with CCT domain	Yano et al., 2000
*Hd6*	*Heading date 6*	Os03g0762000		LD repression	*CKII*	Casein kinase II α subunit	Takahashi et al., 2001; Ogiso et al., 2010
*Hd3a*	*Heading date 3a*	Os06g0157700		SD promotion	*FT*	Similar to phosphatidylethanolamine-binding protein	Kojima et al., 2002
*RFT1*	*Rice FT-like 1*	Os06g0157500		LD promotion	*FT*	Similar to phosphatidylethanolamine-binding protein	Kojima et al., 2002; Ogiso-Tanaka et al., 2013
*Ehd1*	*Early heading date 1*	Os10g0463400	*Ef1*	SD/LD promotion	No obvious ortholog	B-type response regulator	Doi et al., 2004; Saito et al., 2009
*Ghd7*	*Grain number, plant height and heading date 7*	Os07g0261200		LD repression	No obvious ortholog	CCT domain protein	Xue et al., 2008
*DTH8*	*Days to heading on chromosome 8*	Os08g0174500	*Ghd8*/*LHD1*/*Hd5*/*LH8*	SD promotion/LD repression	*HAP3*/*NF-YB*/*CBF-A*	A putative HAP3 subunit of the CCAAT-box-binding transcription factor	Wei et al., 2010; Dai et al., 2012; Fujino et al., 2013; Chen et al., 2014
*DTH3*	*Days to heading on chromosome 3*	Os03g0122600	*OsMADS50*	SD/LD promotion	*SOC1*/*AGL20*	MIKC-type MADS-box protein	Lee et al., 2004; Bian et al., 2011
*Hd17*	*Heading date 17*	Os06g0142600	*Hd3b*/*Ef7*/*OsELF3-1*/*OsELF3*/*Hd-q*	SD/LD promotion	*ELF3*	Similar to ELF3 protein	Matsubara et al., 2012; Saito et al., 2012; Yang et al., 2012; Zhao et al., 2012, 2013
*DTH2*	*Days to heading on chromosome 2*	Os02g0724000		LD promotion	*CO*	CONSTANS-like protein	Wu et al., 2013
*Hd16*	*Heading date 16*	Os03g0793500	*EL1*	LD repression	No obvious ortholog	Casein kinase I	Dai and Xue, 2010; Hori et al., 2013; Kwon et al., 2014
*OsPRR37*	*Oryza sativa Pseudo-Response Regulator37*	Os07g0695100	*Hd2*	LD repression	*PRR7*	Pseudo-Response Regulator	Koo et al., 2013

Locus ID was based on The Rice Annotation Project Database (http://rapdb.dna.affrc.go.jp/).

## Molecular basis of natural variation in rice flowering

A genetic pathway resembling that the photoperiod pathway in *Arabidopsis* [a long-day (LD) plant] is conserved in rice. *Hd1* [a *CONSTANS* (*CO*) homolog in rice] was the first flowering time QTG cloned from natural rice variants. *Hd1* promotes flowering under SD conditions and represses it under LD conditions (Yano et al., 2000) (Figure 1). By contrast, the *Arabidopsis CO* gene promotes flowering under LD conditions (Putterill et al., 1995). The daylength-dependent conversion of Hd1 activity between flowering activator and flowering repressor is caused by phytochrome-mediated signaling (e.g., Ishikawa et al., 2011). The repression of flowering by Hd1 under LD conditions is enhanced by the kinase activity of *Hd6*, which encodes the α-subunit of casein kinase II (Takahashi et al., 2001; Ogiso et al., 2010). *Hd6* enhances the *Hd1* repressor function under LD conditions through the phosphorylation of an unknown protein (Ogiso et al., 2010).

*Hd1* regulates *Hd3a*, a rice homolog of *Arabidopsis FLOWERING LOCUS T* (*FT*) (Kojima et al., 2002) (Figure 1). Tamaki et al. (2007) demonstrated that Hd3a functions as a florigen. Another florigen gene, *RFT1*, is a tandemly duplicated paralog of *Hd3a* (Kojima et al., 2002; Ogiso-Tanaka et al., 2013). Komiya et al. (2008, 2009) found that *RFT1* expression increases under LD conditions, indicating that RFT1 is a LD-specific florigen. More recently, Ogiso-Tanaka et al. (2013) demonstrated that functional defects, which were caused by sequence polymorphisms in the regulatory and coding regions of *RFT1*, contribute to late flowering under LD conditions in an *indica* cultivar.

In *Arabidopsis*, the *CO*–*FT* pathway is regulated by *GIGANTEA* (*GI*), which is a component of the circadian clock (Fowler et al., 1999; Park et al., 1999) (Figure 1). Similarly, regulation of the *Hd1*–*Hd3a* pathway is mediated by *OsGI*, a rice homolog of *GI* (Hayama et al., 2003). These findings reveal that a floral induction pathway from *GI* to *FT* in photoperiodic flowering is conserved between *Arabidopsis* (LD) and rice (SD), but that the photoperiod response differs between these plants.

A unique rice pathway with no obvious ortholog in *Arabidopsis* is also involved in photoperiodic flowering (Figure 1 and Table 1). *Ehd1* is a flowering promoter that encodes a B-type response regulator. *Ehd1* functions upstream of *Hd3a* and *RFT1* (Doi et al., 2004). *Ghd7*, which encodes a CCT (CO, CO-LIKE, and TIMING OF CAB1)-domain protein, was isolated by analysis of natural variations in flowering time. *Ghd7* affects the levels of *Ehd1* and *Hd3a* transcripts, but does not affect *Hd1* mRNA levels (Xue et al., 2008). *Ghd7* represses *Ehd1*, *Hd3a*, and *RFT1* under LD conditions, thereby delaying flowering.

Thus, two independent flowering pathways are present in rice, the conserved *Hd1*–*Hd3a* pathway and a unique *Ghd7*–*Ehd1*–*Hd3a*/*RFT1* pathway, which may integrate environmental photoperiod signals in the control of flowering. In the following sections, we describe additional QTGs that were more recently cloned.

## *Hd17*, a rice homolog of *Arabidopsis ELF3*

Subspecies *japonica* cultivars “Nipponbare” and “Koshihikari” differ in their flowering time and flowering responses to photoperiod. QTL analyses revealed that two QTLs on chromosomes 3 and 6 are involved in the difference in heading date between the two cultivars (Matsubara et al., 2008). The QTL mapped on chromosome 3 was designated as *Hd16*, and the QTL mapped on chromosome 6 was designated as *Hd17*. Both *Hd16* and *Hd17* are involved in photoperiod response, as revealed by observation of heading date in near-isogenic lines (NILs) under SD and LD conditions.

*Hd17* explained a small proportion of the variance in flowering time between “Nipponbare” and “Koshihikari.” Map-based cloning demonstrated that this difference may result in part from a single-nucleotide polymorphism (SNP) within a putative gene encoding a rice homolog of the *Arabidopsis* EARLY FLOWERING 3 (ELF3) protein (Matsubara et al., 2012). The SNP was observed among Asian rice cultivars, mainly in *japonica* cultivars. It seems that the wild-type allele has the “Koshihikari” SNP (i.e., the “Nipponbare” allele is a natural variant), because almost all *indica* cultivars and wild accessions surveyed in the study carry the “Koshihikari” SNP.

The amino acid change (serine to leucine) caused by this SNP in Hd17 may reduce the mRNA level of the floral repressor *Ghd7* under LD conditions, causing ‘Nipponbare’ to flower earlier than NIL-*Hd17*, which carries a chromosomal segment including the “Koshihikari” allele in the “Nipponbare” background. On the other hand, a loss-of-function mutation *ef7* in the rice *ELF3*-like gene (= *Hd17*) seems to increase the *Ghd7* transcription level, and the mutants flower later than wild-type plants under both SD and LD conditions (Saito et al., 2012). This suggests that the *ELF3*-like gene acts as a floral promoter by attenuating the *Ghd7* transcription level (Figure 1).

*Arabidopsis* ELF3 regulates circadian rhythms by affecting the transcription of clock-associated genes such as *LATE ELONGATED HYPOCOTYL* (*LHY*), *CIRCADIAN CLOCK-ASSOCIATED 1* (*CCA1*), *PSEUDO-RESPONSE REGULATORs* (*PRRs*), and *GI*; the clock output gene *CHLOROPHYLL A*/*B BINDING 2* (*CAB2*); and the floral promoter *CO* (reviewed by McClung, 2011) (Figure 1). The circadian clock function is conserved also in rice (Murakami et al., 2007). In the *ef7* mutant, the expression of the luciferase gene driven by the *CAB PROTEIN* (a rice homolog of *Arabidopsis CAB2*) promoter under constant darkness was not affected, but the period of free-running rhythms under constant light was slightly shortened, suggesting that Ef7 mediates light input to the circadian clock but is not required for the clock function in the dark (Saito et al., 2012). By using an *ELF3*-like gene knockout and down-regulation of the expression of *ELF3*-like gene by RNAi, other groups revealed that the *ELF3*-like gene affects the mRNA expression of clock-associated genes and *Hd1* (Yang et al., 2012; Zhao et al., 2012) (Figure 1). These observations suggest that the rice *ELF3*-like gene is also involved in the function of the circadian clock.

## *Hd16*, a gene encoding casein kinase I

The “Koshihikari” allele of *Hd16* decreased photoperiod response in comparison with the “Nipponbare” allele. Map-based cloning revealed an SNP in the gene encoding casein kinase I (CKI), which has no obvious ortholog in *Arabidopsis* (Hori et al., 2013). The SNP resulted in a non-synonymous substitution of an alanine (“Nipponbare”) with threonine (“Koshihikari”). CKI is a protein serine/threonine kinase that is highly conserved among plant and animal species (reviewed by Tuazon and Traugh, 1991; Gross and Anderson, 1998). CKI has various functions in both the cytoplasm and the nucleus, such as DNA repair, and regulation of the cell cycle and circadian rhythm (Liu et al., 2003; Rumpf et al., 2010). Phosphorylation of the clock components by CKI is the key step that initiates and regulates the circadian rhythm. The *tau* gene encodes CKIε in golden hamster (*Mesocricetus auratus*), and a missense mutation in this gene drastically reduces the period of the circadian rhythm (Ralph and Menaker, 1988). The non-synonymous substitution in the “Koshihikari” *Hd16* allele is located at a site close to the *tau* mutation site, and is within the activation loop of the catalytic domain of CKI (Hori et al., 2013). However, the expression patterns of clock-associated genes are similar in the presence of the “Nipponbare” and “Koshihikari” *Hd16* alleles. Therefore, *Hd16* regulates flowering time mediated by the photoperiodic flowering pathway without affecting the regulation of the circadian rhythm.

To reveal the role of *Hd16* in the photoperiodic flowering pathway, we investigated the genetic interactions between *Hd16* and other flowering time QTLs, and the expression levels of the latter (Hori et al., 2013). In rice NILs with functional or deficient alleles of flowering-time genes, significant pairwise interactions were observed between *Hd16* and four other QTLs: *Ghd7*, *Hd1*, *DTH8*, and *Hd2* (*= OsPRR37*). The transcription levels of *Ehd1*, *Hd3a*, and *RFT1* differed between NILs carrying the “Nipponbare” and “Koshihikari” *Hd16* alleles. Biochemical characterization indicated that the Hd16 recombinant protein encoded by the “Nipponbare” allele specifically phosphorylated Ghd7 (but not Hd1) *in vitro*. The kinase activity of “Koshihikari” Hd16 was strongly decreased relative to that of “Nipponbare” (Hori et al., 2013). Thus, Hd16 acts as a Ghd7 inhibitor in the rice flowering-time pathway by enhancing the photoperiod response as a result of Ghd7 phosphorylation (Figure 1).

Another missense mutation was found in the kinase domain of Hd16 in the early-heading Korean cultivar “H143” (Kwon et al., 2014). *In vitro* kinase assays revealed that the “H143” *Hd16* allele is also defective. Thus, there are two defective natural variants of *Hd16*. The “Koshihikari” and “H143” alleles were found only among *japonica* cultivars in temperate areas (Hori et al., 2013; Kwon et al., 2014).

*Hd16* was previously identified as *Early flowering 1* (*EL1*) (Dai and Xue, 2010), which controls rice flowering time by down-regulating the gibberellin (GA) signaling pathway mediated by phosphorylation of SLR1, encoded by *Slender rice 1*. SLR1 phosphorylation suppresses the GA response, whereas Ghd7 phosphorylation enhances the photoperiod response. In both cases, phosphorylation leads to delayed flowering under LD conditions. Thus, *Hd16*/*EL1* appears to be associated with both photoperiod and GA responses in rice flowering.

## Other QTGs underlying natural flowering time variation in rice

In addition to the QTGs described above, four rice homologs of the *Arabidopsis* flowering-related genes have been cloned by using genotypes that show natural variation in flowering time (Table 1). *DTH8* encodes a rice homolog of the *Arabidopsis* HEME ACTIVATOR PROTEIN (YEAST) HOMOLOG 3 subunit of the CCAAT-box-binding transcription factor. Under LD conditions, *DTH8* down-regulates *Ehd1* and its downstream target *Hd3a* and therefore acts as a flowering suppressor (Wei et al., 2010) (Figure 1). A *DTH8* variant also promotes flowering under SD conditions (Yan et al., 2011). Most recently, interaction between DTH8 and Hd1 was demonstrated by yeast-two-hybrid assay (Chen et al., 2014).

*DTH3* encodes a rice homolog of *Arabidopsis* SUPPRESSOR OF OVEREXPRESSION OF CO1, a MIKC-type MADS-box protein. *DTH3* up-regulates *Ehd1* and *RFT1* under both SD and LD conditions and thereby promotes flowering (Bian et al., 2011) (Figure 1). At this QTL, there is functional allelic variation between *O. sativa* and *O. glaberrima*, but probably not among *O. sativa* cultivars.

*DTH2* encodes a CONSTANS-like protein. *DTH2* up-regulates *Hd3a* and *RFT1* under LD conditions and thus promotes flowering (Wu et al., 2013) (Figure 1). *OsPRR37* encodes a rice homolog of *Arabidopsis* PRR7, and down-regulates *Hd3a* expression to suppress flowering under LD conditions (Murakami et al., 2007; Koo et al., 2013) (Figure 1).

## Conservation and diversification of flowering time regulation between rice and *Arabidopsis*

Much progress has been made in understanding the natural genetic variation in rice flowering. 12 QTGs have been isolated and assigned to specific photoperiod flowering pathways. Among them, 9 have homologs in the *Arabidopsis* genome, suggesting that the genetic basis of photoperiodic flowering has an ancient origin in flowering plants (Table 1).

On the other hand, it is evident that there are differences in the photoperiodic flowering pathways between the two species (Figure 1). The *Ghd7*–*Ehd1*–*Hd3a*/*RFT1* pathway, which is modulated by *Hd16*, is not present in *Arabidopsis*. However, in many cases even orthologous genes have divergent functions, as exemplified by *CO* (flowering promotion in LD) and *Hd1* (flowering promotion in SD), and by *ELF3* (flowering repression in SD) and *Hd17* (flowering promotion in both SD and LD). The differences between these orthologs may be associated with neofunctionalization, which is the evolution of new function of a duplicated gene, as suggested about the divergent function of orthologs of *FLOWERING LOCUS VE* and *FT* in sugar beet (*Beta vulgaris*, Pin et al., 2010; Abou-Elwafa et al., 2011). Actually, both *Hd1* and *ELF3* have putative paralog(s) in the rice genome.

Such differences may have resulted from evolution of these lineages in different geographic regions: rice in equatorial regions characterized by stable temperature and daylength all year round, *Arabidopsis* in temperate regions with fluctuating temperatures and changing daylength. As a result, rice has developed photoperiodic flowering, while *Arabidopsis* acquired an additional vernalization requirement as adaptation to the cold season (reviewed by Ballerini and Kramer, 2011).

Probably, this hypothesis should be examined among cereals, such as maize (*Zea mays*), wheat (*Triticum aestivum*) and barley (*Hordeum vulgare*) as well as rice, because they are more closely related to each other as rice and *Arabidopsis* (Magallón and Sanderson, 2005). Additionally, a similar difference in flowering phenology has been observed between tropical-origin (rice and maize) and temperate-origin (wheat and barley) cereals as between rice and *Arabidopsis* (e.g., Greenup et al., 2009; Jung and Müller, 2009).

We suggest that emphasizing divergence among plant species (particularly cereals) rather than conservation would help to better explain the genetic basis of flowering and adaptive divergence in rice.

## Application of natural variation in flowering time genes in breeding programs

Adjustment of photoperiod response changes flowering time and enhances adaptability to local environmental conditions in many plant species, including rice (e.g., Jung and Müller, 2009; Hori et al., 2012). To introduce rice at high latitudes, breeders have selected lines with a weaker response to photoperiod to produce early-flowering cultivars and to ensure maturation during the optimal period (e.g., Izawa, 2007; Ebana et al., 2011). The weak allele of *Hd16* may have permitted the extension of the rice cultivation area into northern regions (Hori et al., 2013; Kwon et al., 2014). Deficient or weak alleles of *Hd1*, *Ghd7*, *DTH8*, *DTH2*, and *OsPRR37* are also distributed in northern rice cultivation areas at high latitudes (Xue et al., 2008; Takahashi et al., 2009; Wei et al., 2010; Koo et al., 2013; Wu et al., 2013), strongly suggesting that such alleles are involved in the expansion of rice cultivation areas.

In addition to several major flowering time QTGs isolated in previous studies, we have identified QTLs with minor effects: *Hd4* (likely allelic to *Ghd7*), *Hd7*, *Hd9* (Lin et al., 2002, 2003), and *Hd17* (Matsubara et al., 2012). These QTLs are necessary for breeders in fine tuning of the flowering time in rice cultivars. Breeders sometimes want to slightly change the flowering time of rice cultivars. Natural variations are observed at many loci (at least more than 20, according to a publicly available database, Q-TARO: http://qtaro.abr.affrc.go.jp/) involved in rice photoperiodic flowering pathways. These variations are available for producing a number of allelic combinations of flowering time QTLs and for developing rice cultivars adjusted to diverse cultivation areas.

## Conclusions and perspectives

Molecular cloning of QTGs underlying natural variation in flowering time of rice has improved our understanding of the genetic basis and provides insights into adaptive evolution and breeding in rice. However, we still know little about the distribution of QTG associated with flowering time in rice cultivars. We are still unable to predict exactly the relative effects of individual QTG on flowering time of rice cultivars in diverse natural field conditions, owing to limited knowledge of QTG × QTG and QTG x environmental interaction. This leads to the gap between genotype and phenotype (discussed by Benfey and Mitchell-Olds, 2008; Olsen and Wendel, 2013). To resolve this problem, we will need to assess the effect of each allele on flowering time in different allele combinations and in various environments by the combination of experimental populations, such as recombinant inbred lines (RILs), chromosome segment substitution lines (CSSLs), nested association mapping (NAM) population and multi-parent advanced generation inter-cross (MAGIC) population, and sequencing the alleles from each line. Furthermore, the transcriptome analysis of flowering-time genes under natural field conditions may also pave the way for the prediction of flowering time (Nagano et al., 2012).

### Conflict of interest statement

The authors declare that the research was conducted in the absence of any commercial or financial relationships that could be construed as a potential conflict of interest.
